# Successional patterns of microbial communities across various stages of leaf litter decomposition in poplar plantations

**DOI:** 10.3389/fmicb.2025.1628355

**Published:** 2025-07-23

**Authors:** Shuang Hu, Bao-Teng Wang, Taihua Li, Su Bu, Chun-Zhi Jin, Long Jin, Hong-Hua Ruan, Kee-Sun Shin, Feng-Jie Jin

**Affiliations:** ^1^College of Ecology and Environment, Co-Innovation Center for Sustainable Forestry in Southern China, Nanjing Forestry University, Nanjing, China; ^2^National Positioning Observation Station of Hung-tse Lake Wetland Ecosystem in Jiangsu Province, Hongze, China; ^3^College of Life Science, Nanjing Forestry University, Nanjing, China; ^4^Korea Research Institute of Bioscience and Biotechnology (KRIBB), Daejeon, Republic of Korea

**Keywords:** poplar litter, phyllospheric microorganisms, microbial community, litter decomposition, chemical property

## Abstract

**Introduction:**

Litter decomposition drives nutrient cycling in terrestrial ecosystems, yet the dynamics of phyllosphere microbial communities during this process remain poorly understood. Poplar leaf litter decomposition is particularly critical due to its widespread plantation use. While prior studies highlight the roles of microbes in decomposition, stage-specific community succession patterns and their driving factors are underexplored. We hypothesize that microbial structure and function correlate with litter nutrient dynamics. This work advances mechanistic insights into poplar litter decomposition and informs sustainable plantation management.

**Methods:**

Poplar leaf litter was sampled periodically during a 342-day decomposition period. DNA was extracted for 16S rRNA gene (bacteria) and ITS region (fungi) high-throughput sequencing. Microbial diversity, composition, and co-occurrence networks were analyzed using QIIME2 and Gephi. Litter quality was measured via elemental analysis and spectrophotometry. Canonical Correlation Analysis (CCA) was used to assess relationships between microbial communities and environmental factors.

**Results:**

The microbial community structure and composition exhibited significant differences at both the class and genus levels throughout the entire decomposition process. Specifically, the dominant fungal taxa, Dothideomycetes, was partially replaced by Sordariomycetes, Tremellomycetes and Leotiomycetes as degradation progressed. Meanwhile, Actinobacteria, Alphaproteobacteria, and Gammaproteobacteria dominated the bacterial communities throughout the entire degradation period, while the abundance of Gammaproteobacteria decreased at later stage and Actinobacteria peaked at t4 stage. Co-correlation networks revealed that the bacterial community had a higher average clustering coefficient and shorter average path lengths compared to fungi, suggesting greater functional diversity and resilience against external disturbances. With the decomposition of leaf litter, the total N content increased gradually, while other nutrients (C, P, K, cellulose and hemicellulose) decreased progressively. Litter characteristics had significant effects on microbial community structure: C/N, TK and residual hemicellulose (RH) were the primary driving factors affecting fungal community structure, whereas bacterial community structure was influenced by TK, RH, residual cellulose (RC) and lignin contents.

**Conclusion:**

Overall, the decomposition of poplar litter is a complex process accompanied by dynamic succession of phyllosphere microbial communities. These results provide insights into the decomposition mechanisms of poplar leaf litter and offer a scientific basis for enhancing nutrient conversion efficiency and productivity of poplar plantations.

## 1 Introduction

Litter is a general term for the organic debris derived from the wilted above-ground plant components in terrestrial ecosystems (Schlesinger and Lichter, 2001). Studies have demonstrated that litter serves as an important global carbon pool, with approximately 90% of aboveground biomass turnover in terrestrial ecosystems occurring in the form of litter in a given year (Cebrian, 1999). The decomposition of litter also serves as a critical pathway for nutrient transfer from plant to soil (María Betania and Natalia, 2021), thereby playing a vital role in regulating biogeochemical cycles and maintaining soil fertility. Previous studies have shown that litter decomposition is influenced by multiple factors, including climate, litter characteristics, microbial communities and the interactions among these elements (Aerts, 1997; Bradford et al., 2016). Among these factors, litter property and decomposer community composition are particularly influential on a small scale (Aerts, 1997; Yu et al., 2023). Studies have shown that differences in C and N stoichiometry between decomposers and available substrates, can drive litter decomposition and nutrient release (Hessen et al., 2004; Parton et al., 2007). Generally, litter quality is categorized into high-quality and low-quality based on nutrient content. High-quality litter typically exhibits lower C/N ratios, greater N content, and lower lignin content, making it more readily decomposed (Bhatnagar et al., 2018; Cornwell et al., 2010). In contrast, low-quality litter is more persistent and difficult to degrade (Castellano et al., 2015). Additionally, lignin concentration has been regarded as a robust predictor of decomposition rates (Zhang et al., 2008). Taylor (Taylor et al., 1989) reported that the lignin-to-nitrogen ratio was associated with litter decomposition in terrestrial ecosystems.

Phyllosphere, defined as the above-ground plant component, constitutes one of the most diverse and widespread habits for microbial life, supporting complex and diverse microbial communities. These inhabitants are referred to as phyllospheric microbes (Lindow and Brandl, 2003). Previous studies have primarily focused on soil microorganisms as the starting point of litter decomposition while largely ignoring the role of phyllosphere microorganisms in this process (Cleveland et al., 2014; Sun et al., 2017). In fact, during the early stages of decomposition, phyllospheric microbes become the first colonizing group in litter and serve as the important participants in litter decomposition by adjusting their life strategies (Pan et al., 2024; Sun et al., 2021). Fanin et al. (2021) indicated that the absence of phyllosphere communities would reduce the home field advantage (HFA) effects.

As the primary decomposers of litter, microorganisms play vital roles in litter decomposition. Traditionally, saprobic fungi have been considered as the main drivers of plant litter decomposition through the secretion of various extracellular enzymes that break down organic matter into smaller molecules (Jia et al., 2021; Takashi et al., 2004). Different fungal taxa exhibit varying abilities to decompose lignocellulose (Leifheit et al., 2024). For instance, many excellent cellulose and hemicellulose degraders have been found to belong to Ascomycota, such as *Penicillium* spp., *Talaromyces* spp., *Trichoderma* spp., and *Aspergillus* spp. (Méndez-Líter et al., 2021; Ogunyewo et al., 2020). Meanwhile, white-rot fungi within the class Agaricomycetes are known for their capacity to break down lignin (Floudas et al., 2012; Treseder and Lennon, 2015). Some studies suggest that bacteria merely colonize the litter surface and rely on readily available substances produced by fungi (Cline and Zak, 2015). However, other researchers argue that bacteria can provide electrons or essential micronutrients to fungi, thus playing an indispensable role in litter decomposition (Frey-Klett et al., 2011). For example, previous studies have suggested that Acidobacteria can degrade lignin (Abdel-Hamid et al., 2013), while *α*-proteobacteria and *β*-proteobacteria have been identified as ligninolytic or capable of degrading cellulose and aromatic structures (Jia et al., 2022; Tian et al., 2014). *Pedobacter* can secrete various enzymes that promote the degradation of cellulose and hemicellulose (Jia et al., 2022). Currently, integrated studies on successional dynamics in both phyllospheric fungal and bacterial communities during litter decomposition remain limited. It is still unclear whether the succession dynamics of fungi align with those of bacteria during the litter decomposition, and their respective contributions to this process are not well understood. Therefore, elucidating the community structure dynamics between fungi and bacteria is undoubtedly valuable for advancing our understanding of litter decomposition.

*Populus* sp. is a widely distributed and fast-growing tree species in China that can thrive in dense plantations (Polle et al., 2013). Litter decomposition plays a crucial role in the ecological nutrient cycle. Therefore, the litter generated from poplar serves as a critical indicator for maintaining energy flow and nutrient cycling within poplar plantations. Based on above information, we selected *Populus* as our research subject and collected litter samples from a plantation located in Jiangsu province, China. We evaluated the dynamic characteristics of litter quality during the decomposition process using the litter bag method. We also explored the diversity and composition of microbial communities through Illumina high-throughput sequencing. In this study, we aim to address the following questions: (1) How do fungal and bacterial communities evolve dynamically during the decomposition of poplar litter? (2) What are the differences in the changes of fungal and bacterial communities during decomposition? (3) How do the chemical properties of litter correlate with the succession of fungal and bacterial communities?

We hypothesized that poplar litter decomposition is a dynamic process regulated by distinct microbial communities at various stages. We further hypothesized that litter characteristics would be closely associated with microbial community composition. We anticipate that our findings will contribute to a deeper understanding of the dynamics of the microbial communities involved in poplar litter decomposition.

## 2 Materials and methods

### 2.1 Sampling site description and experimental design

Our experiments were conducted at a poplar plantation (32° 520’ N, 120° 490′E) located on a forest farm, in Jiangsu province, eastern China. This region has a subtropical climate characterized by a mean annual precipitation of 1,051 mm and a mean temperature of 13.7°C. The soil type is classified as Arenosols with slightly alkaline properties (Yu et al., 2022).

This experiment was initiated in 2020 and involved the establishment of three study units within this pure polar (*Populus deltoides* cv. ‘I-35’) plantation. Our objective was to investigate five time points at different stages of litter decomposition: t0 = freshly fallen litter (November 2020), t1 = 30 days (December 2020), t2 = 121 days (March 2021), t3 = 210 days (June 2021), t4 = 342 days (October 2021). Freshly fallen leaves were collected from the study plots in November 2020 and transported immediately to the laboratory in sterile, sealed polyethylene bags. The material was thoroughly mixed and divided into two parts: one was used as initial litter (t0) for determining moisture content and high-throughput sequencing, while the other was naturally air-dried at room temperature for making litter bags. 10 g of air-dried litter were placed into each nylon mesh bag (20 × 20 cm, mesh size 1 mm × 1 mm). In December 2020, these litter bags were placed back in the top layer of soil in the study plot (Supplementary Figure S1). Then, at each sampling date, four litter bags randomly selected from each study plot were retrieved. These litter samples were divided into two portions: one portion was frozen at −80°C for DNA extraction, while the other was oven-dried at 65°C and ground through a 0.25 mm sieve for chemical analysis. The litter mass residual rate (%)was quantified by calculating the ratio of the dry weight of the remaining litter bags at different sampling time points to the initial dry weight of the litter bags and converting it into a percentage. Ultimately, three biological replicates were set up at each time point (n = 3), resulting in a total of 15 samples across all five time points.

### 2.2 Chemical properties of litter

The concentration of C in leaves was determined using the oil bath K_2_Cr_2_O_7_ titration method (Wang et al., 2021). After digestion of plant samples mainly with H_2_SO_4_-H_2_O_2,_ the concentrations of N, P, and K were determined by traditional methods (Graça et al., 2006; Wang et al., 2021). The P content in litter was examined by the molybdenum-antimony anti-spectrophotometric method (Ba et al., 2020). Cellulose and lignin were quantified using a Content Assay kit (keming, Suzhou, China) according to the manufacturer’s protocol. The hemicellulose content was determined using the 2% hydrochloric acid method (Zhang et al., 2023). Hemicellulose was hydrolyzed into xylose by heating under acidic conditions, and the released xylose content was then measured to calculate hemicellulose content. All these experiments were biologically repeated three times.

### 2.3 Genomic DNA extraction and Illumina MiSeq sequencing

Total genomic DNA was extracted using the HiPure Soil DNA Kits (Magen, Guangzhou, China) according to the manufacturer’s protocols. The primers 341F (CCTACGGGNGGCWGCAG) and 806R (GGACTACHVGGGTATCTAAT) were used to amplify the V3-V4 region of bacterial 16S rRNA gene, while the universal primers ITS3_KYO2 (GATGAAGAACGYAGYRAA) and ITS4 (TCCTCCGCTTATTGATATGC) was used to amplify the fungal internal transcribed spacer 2 (ITS2) region. The PCR amplification condition was as follows: initial denaturation at 95°C for 5 min, followed by 30 cycles of denaturation at 95°C for 1 min, annealing at 60°C for 1 min, and extension at 72°C for 1 min, with a final extension at 72°C for 7 min. The PCR amplicons were purified, quantified, and pooled for subsequent sequencing on the Illumina Novaseq 6,000 platform. Raw sequencing data have been deposited in the National Center for Biotechnology Information (NCBI) Sequence Read Archive (SRA) with accession numbers PRJNA1199174 and PRJNA1202579 for bacteria and fungi, respectively.

### 2.4 qPCR analysis

Real-time quantitative PCR analysis was conducted to quantify the copy numbers of the bacteria16S rRNA gene and the fungal ITS gene. Microbial DNA was extracted from 0.2000 g of leaf litter using the FastDNA™ Spin Kit for Soil (MP Biomedicals, Solon, OH, USA). The extracted DNA was then quantified using a NanoDrop 2000c Spectrophotometer (NanoDrop Technologies, Wilmington, DE, USA). The amplification was performed using the StepOneTM Real-Time System (Applied Biosystems, Thermo Fisher, Waltham, MA, USA). The primer sequences used for qRT-PCR were detailed in Supplementary Table S1. The reaction mixture consisted of 1 μL forward primer, 1 μL reverse primer, 15 ng DNA template, 10 μL of 2x SYBR Green qPCR Mix (Accurate Biotechnology, Hunan, China), and 0.4 μL ROX reference dye, with nuclease-free water added to a final volume of 20 μL. Amplification was carried out according to the manufacturer’s instructions. Standard curves were generated using serial dilutions (10^^3^–10^^9^) of purified amplicons, showing a linear relationship between the log gene copy number and the calculated threshold (Ct) value (R^2^ > 0.99). *Agrobacterium radiobacter* was used for bacterial amplifications (172 bp amplicon), and *Aspergillus fumigatus* for fungal amplifications (265 bp amplicon). The number of gene copies in all samples was calculated based on these standard curves. Amplification efficiencies ranged from 98 to 106%. All samples and standards were analyzed in triplicates, with each run including a negative control. The obtained results were then converted to copy numbers per gram of litter.

### 2.5 Bioinformatics and statistical analysis

Sequence data processing and analysis mainly used QIIME2 (2021.04), and the UCHIME algorithm was employed to identify and remove chimeric sequences (Edgar et al., 2011). Sequences exhibiting 100% similarity were clustered into identical amplicon sequence variants (ASVs). Alpha diversity was evaluated using the chao1 and Shannon indices. The Chao1 index represented the observed number of ASVs in the samples, reflecting species richness. The Shannon index was utilized to assess species diversity. Two indices were computed by QIIME2 (2021.04) (Caporaso et al., 2010). The relative abundance of each taxonomic group was visualized using Krona (version 2.6) (Ondov et al., 2011). Nonmetric multidimensional scaling (NMDS) analysis based on Bray–Curtis dissimilarities was conducted to illustrate the patterns of fungal and bacterial community structure at different stages. Analysis of similarities (ANOSIM) was used to determine the intergroup differences. Canonical Correlation Analysis (CCA) was applied to elucidate the influence of environmental factors. SPSS (ver 27.0) software was used to analyze the physicochemical properties of litter during the decomposition process. One-way analysis of variance followed by Tukey’s HSD *post hoc* test was employed to compare the differences among treatments, with *p* < 0.05 considered statistically significant. In addition, Gephi was used to examine and visualize microbial community co-correlation networks, analyzing changes in fungal and bacterial communities. We also calculated the parameters of co-correlation networks, such as the average degrees, average path lengths, network densities, average clustering coefficients, and network diameters (Jiao et al., 2020). In this network, the higher average degrees, network densities and clustering coefficient, the closer the connectivity of network (Ma et al., 2016).

## 3 Results

### 3.1 Litter chemical properties throughout the decomposition process

**Figure 1:**
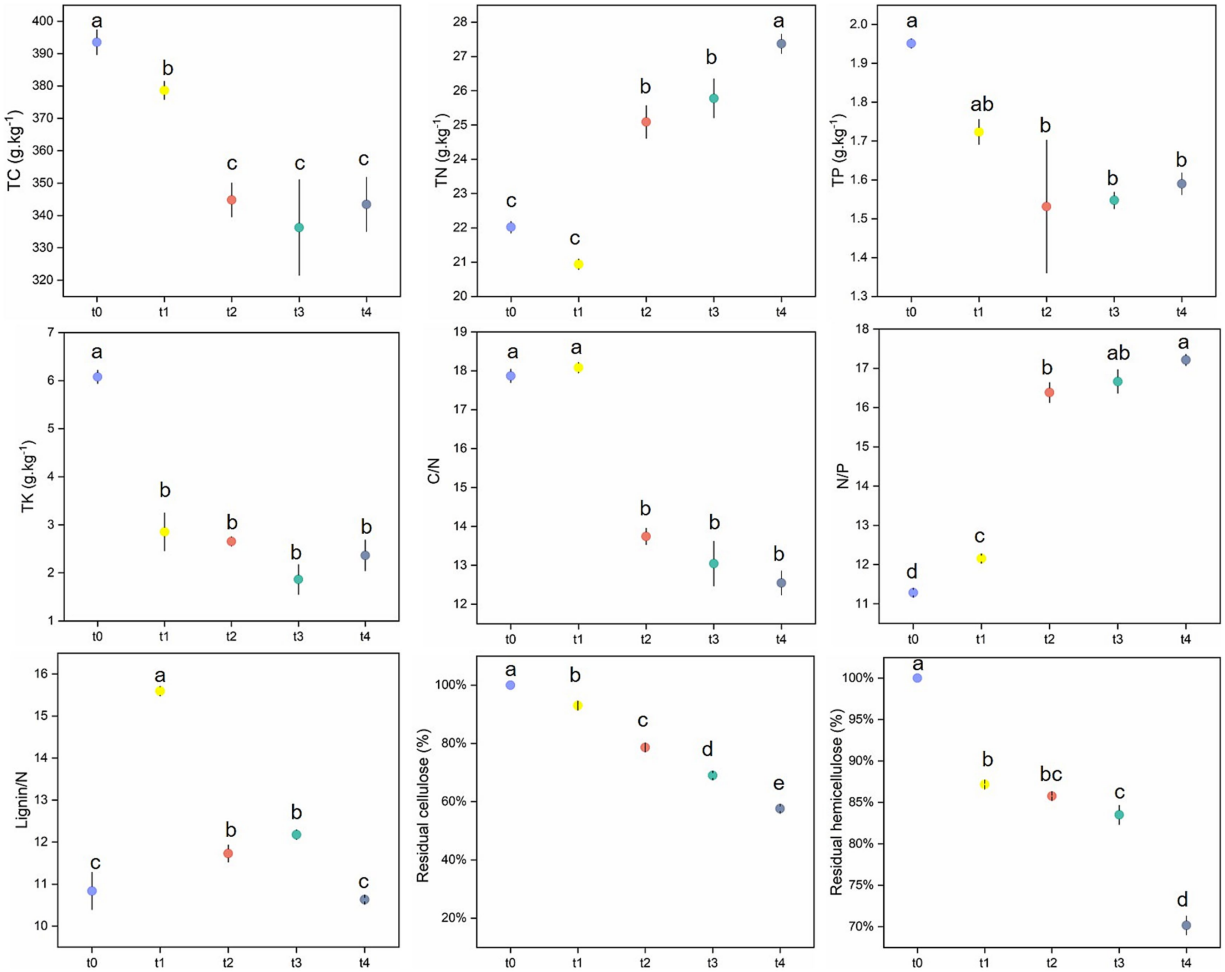
Changes of litter chemical properties at different stages.

Mass measurement results showed that the mass of leaf litter decreased significantly as decomposition progressed. After 342-day decomposition, the residual rate of leaf litter was 72.2% (Supplementary Figure S2), which had significant effect on the content of most nutrients (Figure 1; Supplementary Table S2). During the degradation process of poplar litter, the content of total nitrogen (TN) increased over time and peaked at the t4 stage. In contrast, the contents of other nutrients, such as total carbon (TC), total phosphorus (TP), and total potassium (TK), decreased significantly throughout the decomposition process (Figure 1). Specifically, the contents of TC and TK in litters were highest at the initial stage (t0), decreased significantly at stage t1, and reached the lowest point at stage t3. TP content was also highest at t0 but began to decline significantly from t2 onwards. The C/N ratio exhibited a peak at t1 before decreasing markedly in subsequent stages. Meanwhile, the N/P ratio increased significantly during decomposition, reaching its maximum at t4. Throughout the decomposition process, the residual cellulose and hemicellulose levels declined substantially, while the lignin/N ratio initially increased at t1 before decreasing significantly thereafter.

### 3.2 *α*-diversity of microbial communities

**Figure 2:**
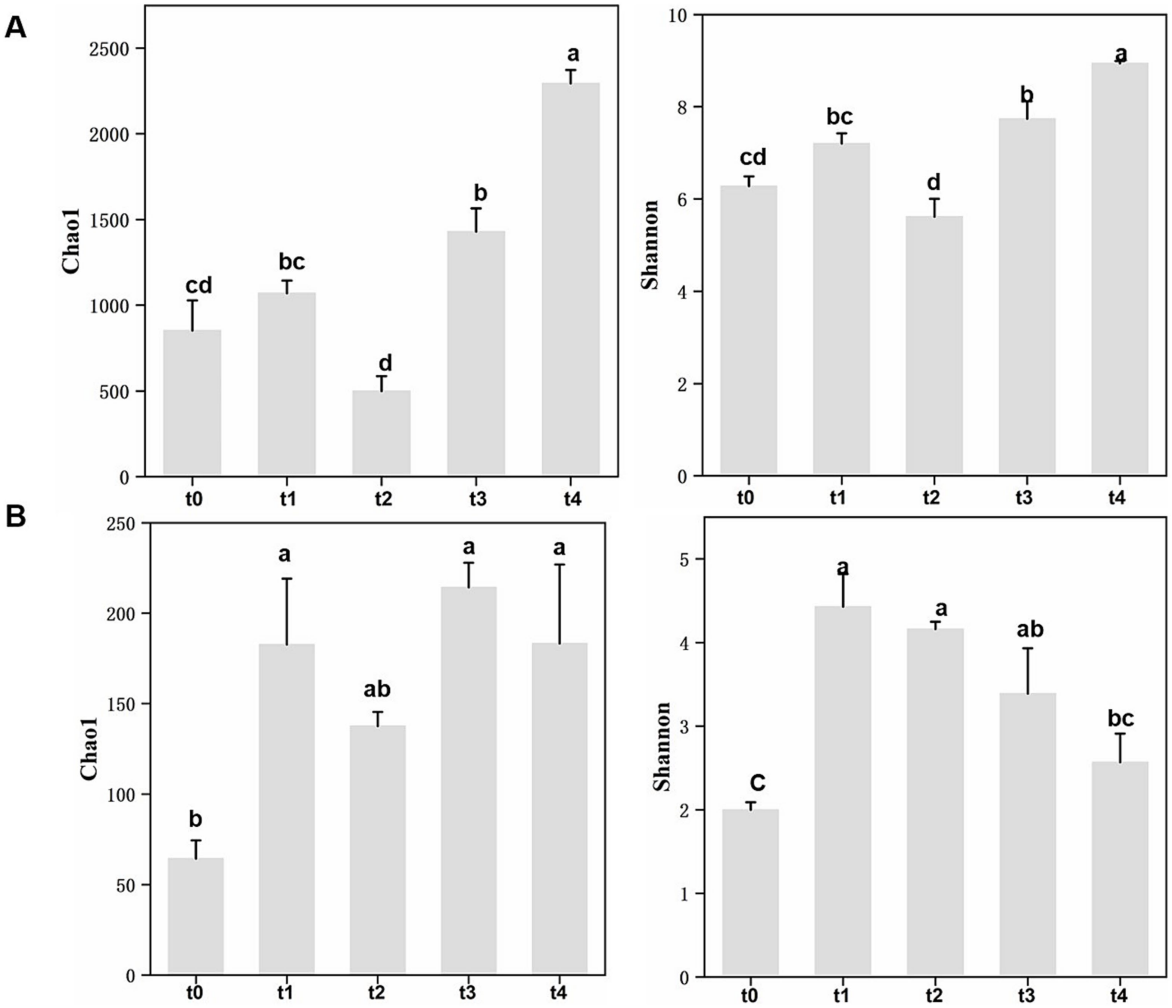
Analysis of *α*-diversity of microbial communities at different stages of leaf litter degradation. **(A)** Chao1 and Shannon indices for bacterial communities. **(B)** Chao1 and Shannon indices for fungal communities. Different lowercase letters indicate significant differences between different stages with Tukey’s test at the significant level of 0.05. t0-t4 represent five time points for litter decomposition: t0 = freshly fallen litter, t1 = 30 days, t2 = 121 days, t3 = 210 days, t4 = 342 days.

A total of 861 fungal amplicon sequence variants (ASVs) and 13,340 bacterial ASVs were identified in this study based on 100% sequence similarity. For the bacterial communities, multiple indexes of α-diversity showed an overall upward trend, with Shannon and Chao1 indices reaching their highest values at the t4 stage and lowest at the t2 stage (Figure 2A; Supplementary Table S3). In contrast, for the fungal communities, both Shannon diversity and Chao1 index were lowest at t0, considerably rose at t1, and subsequently, the Shannon index gradually dropped, reaching a significant fall by t4 (Figure 2B; Supplementary Table S3). Overall, the diversity of bacterial and fungal communities showed an opposite trend during litter degradation: Shannon diversity and richness of bacterial communities progressively increased, while fungal Shannon diversity initially rose sharply but then gradually declined.

### 3.3 Microbial community composition and relative abundance across different stages of litter decomposition

**Figure 3:**
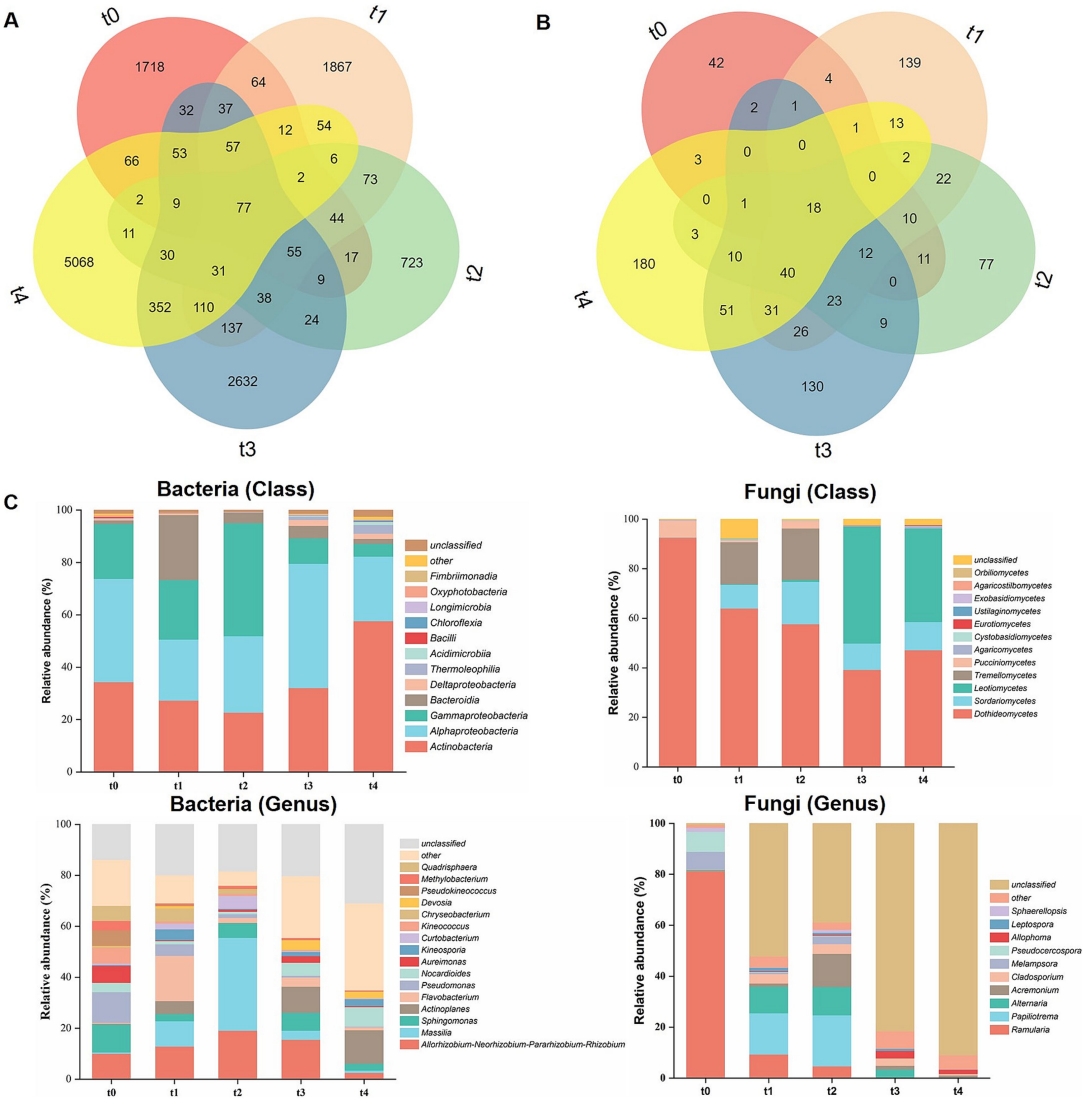
Comparison of microbial community composition at different stages of litter decomposition. Venn diagram analysis of overlapping and unique ASVs numbers of bacterial **(A)** and fungal **(B)** communities at different degradation stages. **(C)** Composition of bacterial and fungal communities at class and genus levels. Each bar represents the percentage mean value for each stage.

To gain a more comprehensive understanding of the microbial community succession patterns during litter decomposition, we also investigate the changes in microbial community composition. A Venn diagram was employed to illustrate the common and unique ASVs across each sample during the decomposition process (Figures 3A,B). Specifically, the number of shared bacterial ASVs was 77, while the number of common fungal ASVs was 18. The number of unique ASVs varied among time points, with the bacterial community showing a pattern of t4 > t3 > t1 > t0 > t2, and the fungal community showing t4 > t1 > t3 > t2 > t0.

**Figure 4:**
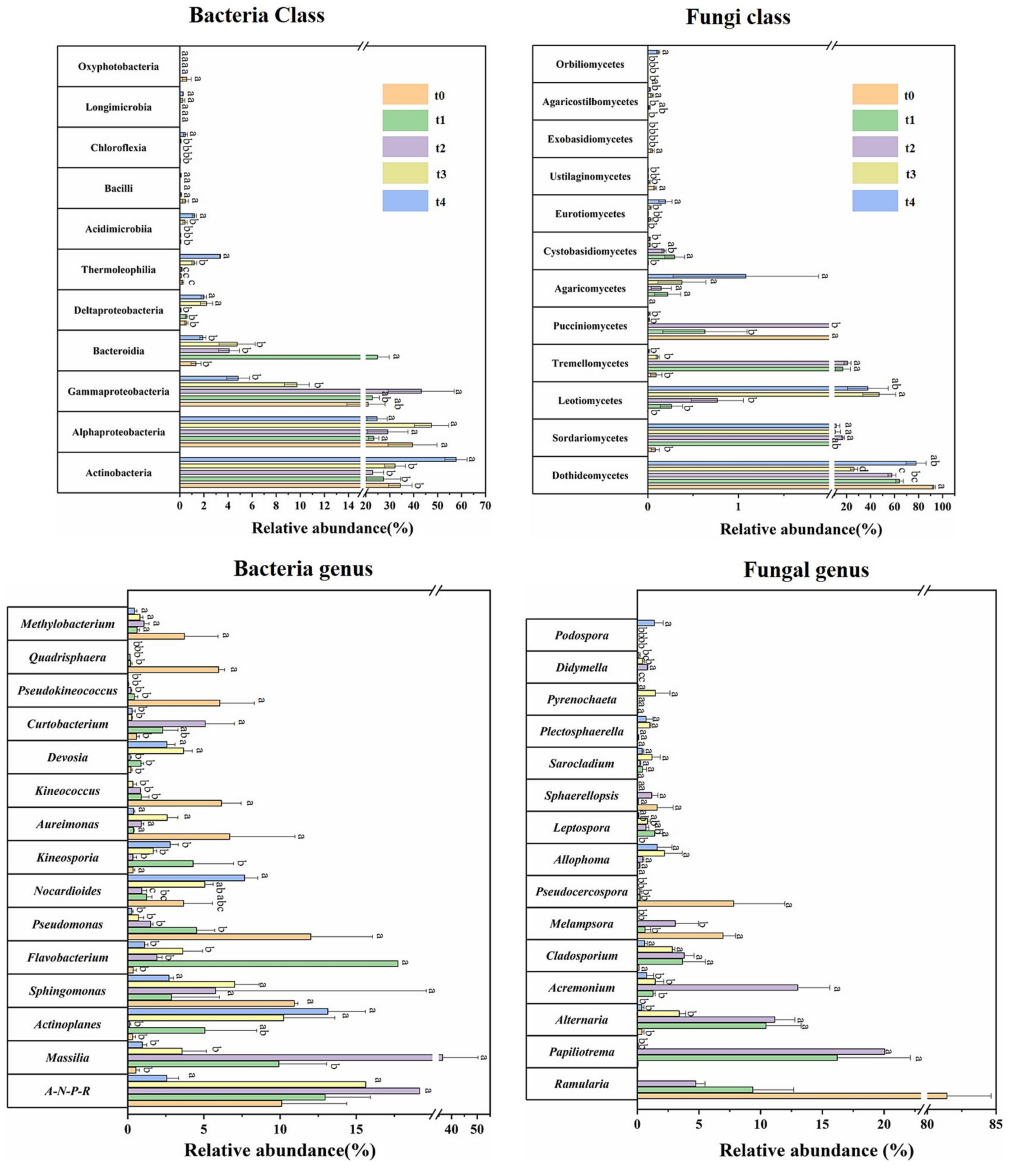
Relative abundances of microbial community composition during different stages of litter degradation. Relative abundances of major bacterial and fungal taxa at class and genus levels. Different lowercase letters denote significant differences among decomposition stages as determined by Tukey-test at the 0.05 significance level.

The composition and relative abundance of microbial communities were further investigated (Figures 3C, 4; Supplementary Table S4–S7). At the class level, bacterial communities were consistently dominated by Actinobacteria (22.80–57.69%), Alphaproteobacteria (23.31–47.48%) and Gammaproteobacteria (4.84–22.73%) across all stages of decomposition (Figures 3C, 4; Supplementary Table S4). The relative abundance of Bacteroidia significantly increased at the t1 stage, becoming a dominant group. Additionally, the relative abundance of Actinobacteria and Gammaproteobacteria peaked at the t4 and t2 stages, respectively, in which the latter dropped rapidly after that. In addition to these, the relative abundance of Deltaproteobacteria, Thermoleophilia, and Acidimicrobiia also peaked at the late stage of degradation. Ascomycota and Basidiomycota were the predominant fungal phyla throughout all stages of litter decomposition. At the class level, fungal communities exhibited distinct changes as decomposition progressed (Figures 3C, 4; Supplementary Table S5). Dothideomycetes was the predominant class at the t0 stage, with a relatively abundance of 92.59%, which decreased significantly as decomposition advanced but rebounded in the last t4 stage. In the t1 and t2 stages, the dominant position of Dothideomycetes was partially replaced by Tremellomycetes and Sordariomycetes. The relative abundance of Leotiomycetes significantly increased at the t3 stage. Leotiomycetes, Dothideomycetes and Sordariomycetes dominated fungal communities at the t3 and t4 stages (Figures 3C, 4; Supplementary Table S5), with Leotiomycetes reaching its highest dominance at the t3 stage, accounting for 47.17% of the community.

Differences in bacterial community composition across different stages was also significant at the genus level (Figures 3C, 4; Supplementary Table S6). *Allorhizobium*-*Neorhizobium*-*Pararhizobium*-*Rhizobium* (*A-N-P-R*), *Massilia* and *Sphingomonas* were dominant during the first four stages. *Flavobacterium* abundance reached its peak at the t1 stage and significant decreased as decomposition progressed. *Massilia* dominated from the t1 to t3 stages, and peaked at the t2 stage. *Pseudomonas* abundance peaked at the t0 stage and decreased as decomposition progressed. *Actinoplanes* abundance significantly increased at later stages (t3 and t4). For fungal community, *Ramularia* (81.42%) was the most dominant fungal genus at the t0 stage, and its abundance decreased sharply as litter decomposition progressed. *Papiliotrema* (20.04%) and *Alternaria* (11.14%) became the dominant genera at the t1 and t2 stages, reaching their maximum proportions at the t2 stage (Figures 3C, 4; Supplementary Table S7). These results suggest that the dynamic changes in bacterial and fungal communities are substantial at both the class and genus levels throughout the entire process of litter degradation.

NMDS analysis showed that poplar litter decomposition significantly affected bacterial and fungal community structure. Specifically, samples from the t0, t3, and t4 stages were distinctly separated from each other, and they were also different clearly from t1 and t2stages. In contrast, samples from t1 and t2 were indistinguishable from one another (Supplementary Figure S3).

### 3.4 Correlations among microbial communities during litter decomposition

**Figure 5:**
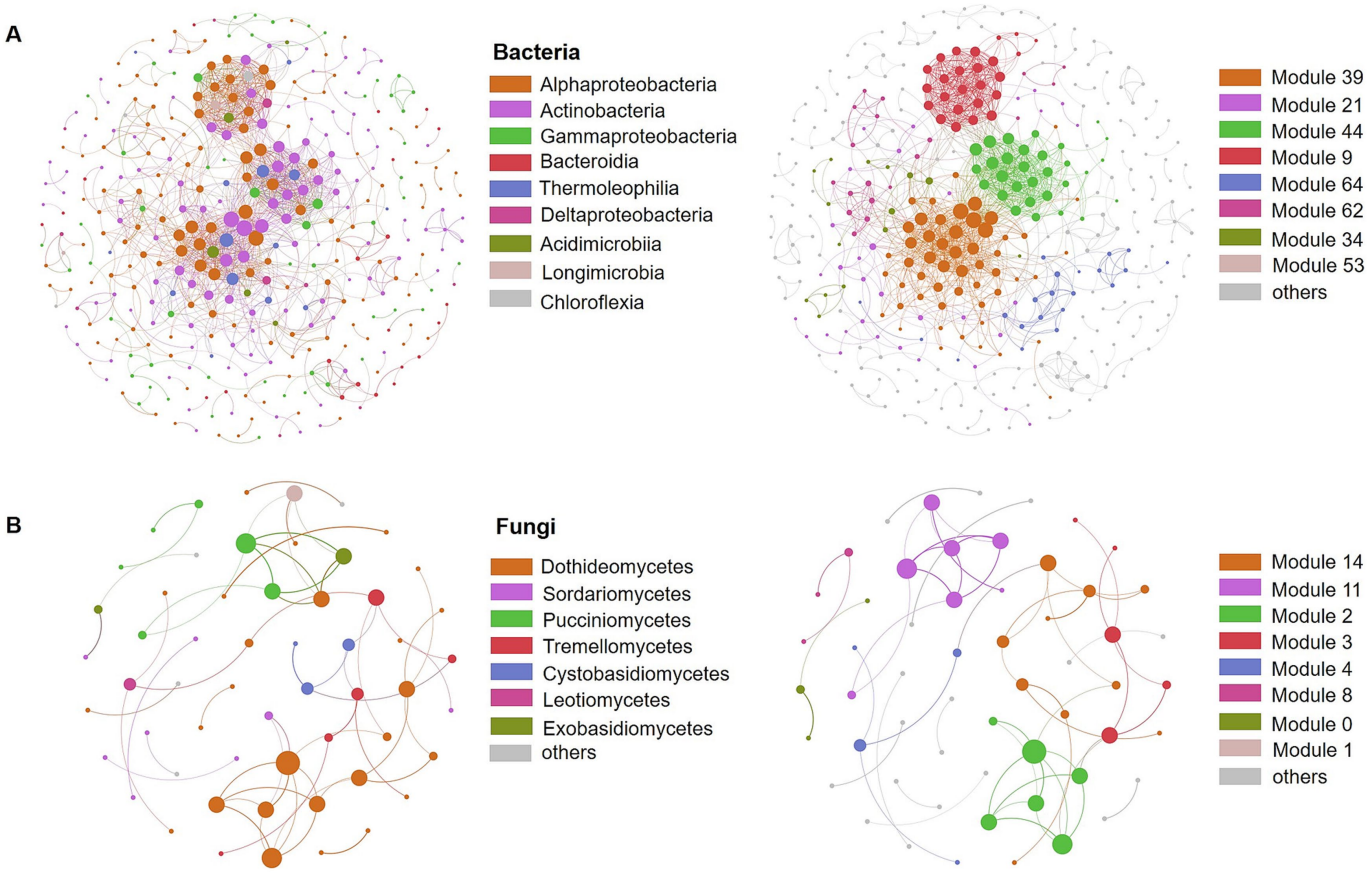
Co-correlation networks of phyllosphere bacterial **(A)** and fungal **(B)** community composition. The colors of the nodes represent the main class (left) or module (right), respectively.

We conducted co-correlation analysis of the microbial community to assess the types of interactions among the bacterial and fungal communities during the litter decomposition process (Figure 5). The results indicated that the bacterial network comprised 372 nodes and 1,363 edges, while the fungal network contained only 56 nodes and 58edges (Figures 5A,B; Supplementary Table S8). Network properties reflect the connectivity and interaction within the microbial community. In this study, the bacterial network exhibited higher average degree and average clustering coefficient, as well as a shorter average shortest path length compared to the fungal network, suggesting that the connectivity among the fungal community was relatively weaker than that of the bacteria community (Supplementary Table S8). The modularity values, which reflect the modular structure of the networks, were 0.667 for bacteria and 0.788 for fungi. A total of 66 modules were identified in the bacterial network and 14 in the fungal network. Further analysis revealed that Alphaproteobacteria, Actinobacteria, and Gammaproteobacteria accounted for more than 80% of the nodes in the bacterial network, while Dothideomycetes constituted 50% of the nodes in the fungal network, consistent with their relatively high abundance in the microbial community (Figure 5).

### 3.5 Relationships between microbial communities and litter chemical properties

**Figure 6:**
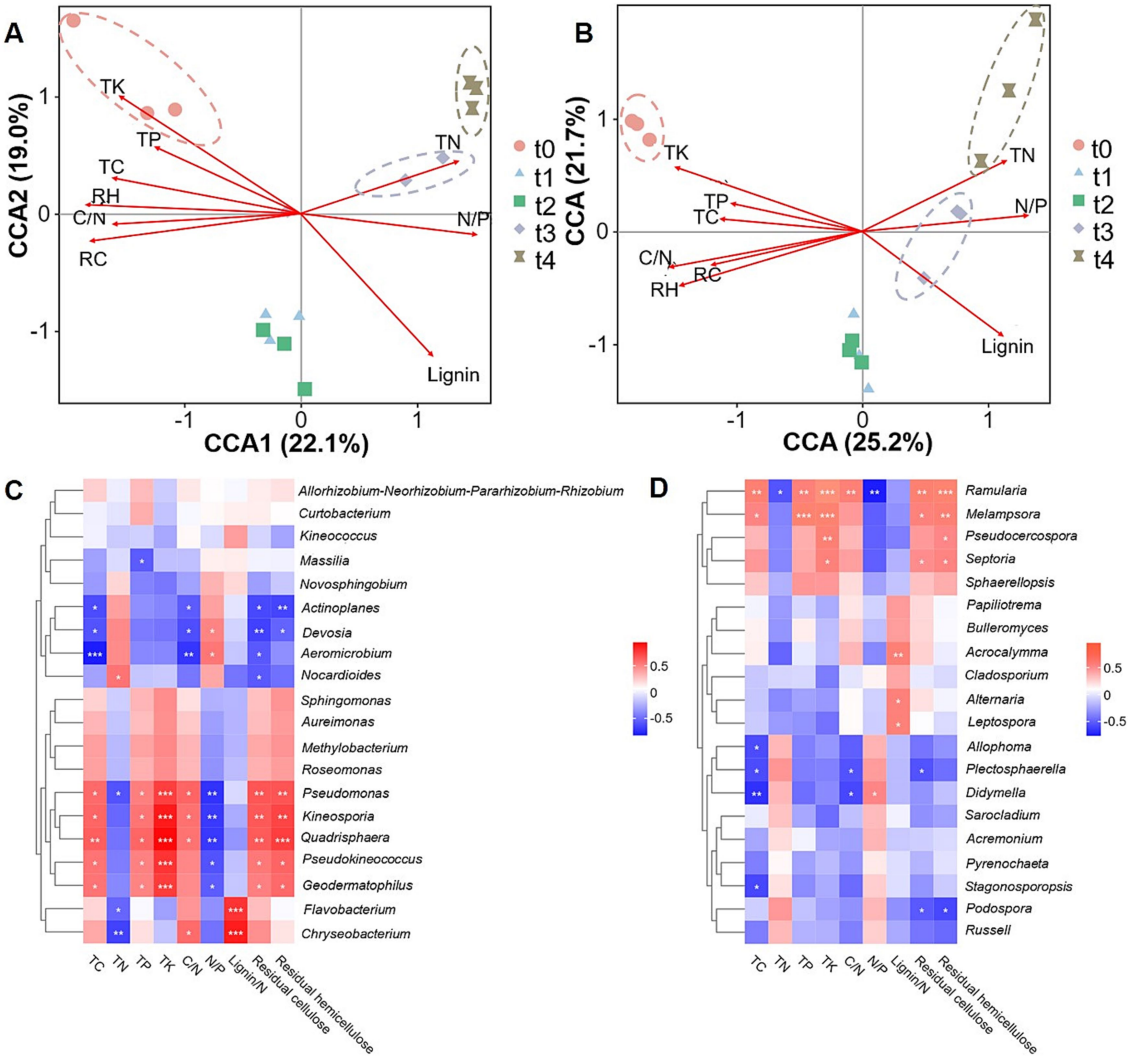
Correlation analysis between microbial communities and litter properties during leaf litter degradation. Canonical Correlation Analysis (CCA) between bacterial **(A)** or fungal **(B)** communities and litter properties. RC: Residual cellulose. RH: Residual hemicellulose. **(C)** Heatmap analysis of correlations between dominant bacterial genera and litter properties. **(D)** Heatmap analysis of correlations between dominant fungal genera and litter properties. Asterisks (*, **, and ***) denote statistical significance levels of *p* < 0.05, *p* < 0.01 and *p* < 0.001, respectively.

To evaluate the extent to which litter properties influence the composition and structure of microbial communities, we conducted a Canonical Correlation Analysis (CCA) on various variables (Figures 6A,B). The results showed that TK, TC, C/N, residual cellulose (RC), residual hemicellulose (RH), and TP were positively correlated with the microbial community composition at t0 stage, and negatively correlated with the microbial composition at t3 and t4 stages. In contrast, TN and N/P were positively correlated with microbial community composition at t3 and t4 stages (Figures 6A,B). Among them, TK, lignin, RC and RH mainly affected the bacterial community structure of litter (Figure 6A), while the contents of lignin, TK, C/N and RH were dominant chemical factors influencing fungal community structure during litter decomposition (Figure 6B).

A further correlation analysis between microbial genera and litter properties showed that: the dominant bacterial genera *Pseudomonas*, *Kineosporia*, *Quadrisphaera*, *Pseudokineococcus,* and *Geodermatophilus* were significantly and positively correlated with TK, RC, RH and TC content, indicating their significant role in the degradation of cellulose and hemicellulose. *Flavobacterium* and *Chryseobacterium* exhibited a significantly positive correlation with lignin/N ratio, but were negatively correlated with TN. *Actinoplanes*, *Aeromicrobium*, and *Devosia* displayed a significantly negative correlation with TC, C/N, and RC (Figure 6C). For fungal community, the dominant genera *Ramularia*, *Melampsora,* and *Pseudocercospora* were significantly and positively correlated with TK and RH in litter, and the former two genera were also positively correlated with TC, TP and RC. In addition, *Acrocalymma*, *Alternaria* and *Leptospora* exhibited positive correlations with lignin/N, while *Allophoma*, *Plectosphaerella*, *Stagonosporopsis*, and *Didymella* were negatively correlated with TC (Figure 6D).

### 3.6 Changes of gene copy numbers in bacterial and fungal communities during litter decomposition

**Figure 7:**
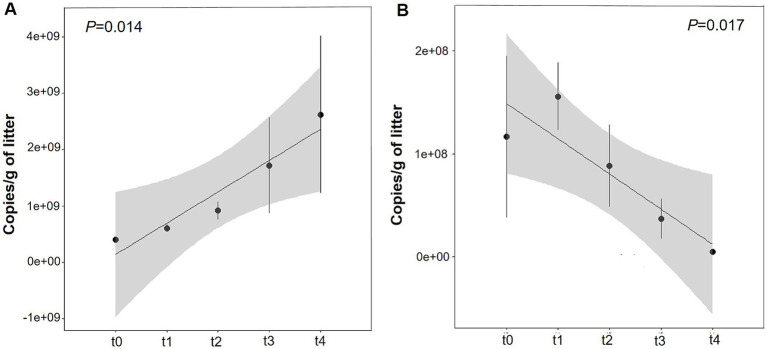
Quantitative analysis of microbial gene copy number variations across different litter decomposition stages. **(A)** Bacteria **(B)** fungi.

The copy numbers of bacterial 16S rRNA gene and fungal ITS gene were quantitatively analyzed by qPCR. Throughout the entire litter degradation process, the copy number of bacterial 16S rRNA gene was significantly influenced by decomposition time (*p* < 0.014) and increased significantly from t0 to t4, reaching its peak at stage t4 (Figure 7A; Supplementary Figure S4A). In contrast, the copy number of the fungal ITS gene was highest at stage t1 and decreased markedly with the decomposition of litters (Figure 7B; Supplementary Figures S4A,B). These results suggest that decomposition time has significant effect on copy number of bacteria and fungi. Fungal communities potentially play a critical role in the early and middle stages of litter degradation, whereas bacterial communities assume greater importance in the middle and late stages.

## 4 Discussion

### 4.1 Characteristics of nutrient release during the litter decomposition process

Litter mass refers to the physicochemical composition of litter, which includes both easily degradable components and resistant fractions. Strict stoichiometric requirements are essential for microbial growth; thus, the scarcity of a particular nutrient may result in a lower decomposition rate of litter (Barantal et al., 2014; Ni et al., 2021). Consequently, litter quality is considered one of the primary controlling factors affecting the litter decomposition rate (Cornwell et al., 2010). C, N, P are key elements involved in litter decomposition, with most research focusing on the quality of the litter matrix and their release dynamics (Carrasco-Barea et al., 2022; Wei et al., 2020). The litter decomposition rate is positively correlated to N and P content but negatively correlated with TC, cellulose and lignin contents (Cao et al., 2016). C/N and N/P ratios have been widely accepted as common indicators for predicting litter decomposition rate (Gautam et al., 2016; Tie et al., 2022). Generally, a lower C/N ratio is associated with a faster decomposition rate (Enríquez et al., 1993). Studies have shown that C/N ratio is significantly reduced and N/P is increased with decomposition, which is consistent with the results of this study (Supplementary Table S2), indicating that C and P were preferentially released over N (Carrasco-Barea et al., 2022; Xie et al., 2024). Furthermore, litter N/P can serve as an indicator of nutrient limitation, where N/*p* > 25 suggests P limitation on litter decomposition (Liu et al., 2016). In this study, although the N/P ratio increased during litter decomposition, it remained below 25 (Figure 1), indicating that poplar litter decomposition was not limited by P content during our experimental period. Additionally, other specific chemical compositions in litter, such as polysaccharides (cellulose, hemicellulose), and phenolic compounds (such as lignin), also influence litter decomposition (Duan et al., 2018). In this study, both fungal and bacterial community were significantly correlated with the amount of cellulose, hemicellulose and lignin (Figure 6).

### 4.2 Changes in microbial community composition and structure during litter decomposition

Our results demonstrated that bacterial Shannon diversity and richness increased progressively with the degradation of litters and reached a peak value at t4 (Figure 2). Meanwhile, bacterial 16S rRNA gene copy number also showed a significantly rise (Figure 7A). These trends are consistent with observations made by Tláskal et al. (2016). In contrast, fungal Shannon diversity and ITS copy numbers, as confirmed by qPCR, both peaked at t1 (Figures 2B, 7B) and subsequently declined, suggesting that the fungal community played a more important role in the early stages of degradation (Baldrian, 2017).

Furthermore, prior research has confirmed the presence of a core functional cluster within the phyllospere that exerts a synergistic effect on litter decomposition (Ruiz-Pérez et al., 2016; Sun et al., 2021). In this study, we also identified the core functional groups (18 fungal and 77 bacterial ASVs; Figures 3A,B) that remained present throughout the decomposition stages. These findings further supported the existence of core functional taxa during litter decomposition.

In general, fungi and bacteria collaborate to expedite litter decomposition (Purahong et al., 2016; Wright, 2005). In this study, Ascomycetes are the dominant phylum in the early stages of litter decomposition and serve as an early decomposers, which is consistent with the previous research results (Jia et al., 2022; Voříšková, 2013). For bacterial community, Proteobacteria and Actinobacteria were predominant across all samples at different time points, both classes are well-known as lignin degraders (de Gonzalo et al., 2016; Granja-Travez et al., 2020; Lee et al., 2019). Meanwhile, Proteobacteria also have a prominent ability to break down proteins (Kazakov et al., 2009).

During the litter decomposition process, microbial communities undergo more pronounced succession at a lower taxonomic level, particularly at the genus level. Genera such as *Sphingomonas*, *Pseudomonas*, *Massilia*, *Mythylobacterium*, *Kineosporia*, and *Flavobacterium* were common phyllosphere bacteria that could be isolated from plant debris (Janakiev et al., 2022). In this study, some of these genera exhibited relatively high abundance during both the early and later stages of litter decomposition, consistent with previous studies reporting their ability to utilize complex substrates or fix nitrogen (Purahong et al., 2016). This may be attributed to improve nitrogen availability and promote microbial growth, potentially explaining increase in nitrogen content during decomposition. Moreover, different bacterial genera play diverse roles in the litter decomposition process. For example, *Curtobacterium* has the potential to degrade structural polysaccharides and release arsenic from litter (Chase et al., 2016; Wang et al., 2024). Regarding the fungal community, Dothideomycetes is a dominant taxon in the phyllospheric fungal community and secretes a variety of cellulases and hemicellulases (Sun et al., 2021; Wang et al., 2020), which predominated during the early stage but gradually decreased as litter decomposition progressed (Figures 3C, 4). The gradual decomposition of cellulose, soluble starch, and other polysaccharides led to an accumulation of lignin, favoring the colonization of Leotiomycetes and Sordariomycetes during later decomposition stages (Liu et al., 2024). At the genus level, although *Ramularia* was dominant at the initial stage (t0), it was quickly replaced by *Cladosporium*, *Alternaria*, *Acremonium*, and *Papiliotrema* (Figures 3C, 4). The genus *Papiliotrema* is commonly found in the phyllosphere (Surussawadee et al., 2014), while *Cladosporium* and *Alternaria* are widely distributed in nature and can utilize small-sized molecular sugars and starch (Carrasco-Barea et al., 2022). Earlier findings supported that *Acremonium* exhibits high CMCase and FPA activity, suggesting its significant role in early litter decomposition (Hao et al., 2006).

### 4.3 Microbial interaction during litter decomposition

Correlation network analysis enabled us not only examine the composition of the microbial community, but also deepen our understanding of the ecological interactions between different species and how these interactions are influenced by driving factors (Montoya et al., 2006). The parameters of network properties indicated that the bacterial network is more complex, with higher connectivity and interaction compared to the fungal network (Figure 5). This suggests that bacteria have greater resource abundance, higher information transmission rates, and higher functional diversity than fungi (Wagg et al., 2019). Additionally, it can be inferred that the bacterial community exhibited higher tolerance to environmental disturbances due to its network complexity (Wang et al., 2018). In this study, taxa with high abundance were found to contain more nodes in the ecological network, indicating that species with relatively high abundance also possess high interaction potential within the community. Overall, our results revealed that bacteria were more susceptible to external environmental changes. Given their rapid succession during litter decomposition, this finding further supports the significant role of bacteria in this process (Jia et al., 2022).

### 4.4 Relationship between succession of microbial communities and changes in litter quality

Numerous environmental factors have been documented to influence microbial community succession during the litter decomposition process (Jia et al., 2022; Jia et al., 2021). This study also demonstrated that the nutrient content in litter was significantly correlated with both fungal and bacterial communities. Previous research has shown that lignin content is significantly associated with bacterial communities (Chen et al., 2021). Our results further revealed significant correlations between residual cellulose, residual hemicellulose, and bacterial communities, indicating the substantial decomposing capabilities of these microorganisms. C/N ratio and lignin content were critical factors driving fungal community succession. Similar results have been observed in other studies (Chen et al., 2021). K is essential for microbial growth and reproduction (Purahong et al., 2016). Higher K content has been reported to increase the relative abundance of beneficial bacterial groups involved in nitrogen fixation, phosphorus dissolution, and potassium mobilization (Lu et al., 2022). According to our results, K content in litter was significantly correlated with both fungal and bacterial communities.

## 5 Conclusion

In this study, we investigated the dynamic succession of phyllospheric fungal and bacterial communities during the decomposition of poplar leaf litter. Our results indicated that the microbial community composition and structure exhibited significant variation at different stages of decomposition. Meanwhile, the network of bacterial communities demonstrated closer interactions and a higher degree of segregation than fungal network, suggesting greater functional diversity and stability in response to external interference. We also observed that the nutrient content in the litter (including cellulose, hemicellulose, P, C, and N) significantly influenced the dynamics of the phyllospheric microbial community during litter decomposition. This study provides deeper insights into the role of microorganisms in litter decomposition and offers valuable perspectives on the processes within plantation forest ecosystems and nutrient cycling. Furthermore, it has potential applications for assessing future global carbon dynamics and monitoring forest ecosystem stability. However, several limitations should be noted. Due to geographic specificity, the observed patterns may not fully apply to other ecosystems with distinct climatic or edaphic conditions. Meanwhile, while litterbags standardized the decomposition measurements, their mesh size may selectively exclude certain microbes or soil fauna, potentially altering decomposition rates compared to natural litter layers.

## Data Availability

The datasets presented in this study can be found in online repositories. The names of the repository/repositories and accession number(s) can be found in the article/Supplementary material.
