# Supplementary material for: Successional patterns of microbial communities across various stages of leaf litter decomposition in poplar plantations
Source: Front Microbiol. 2025 Jul 23;16:1628355. doi: 10.3389/fmicb.2025.1628355 (PMC12325270; doi:10.3389/fmicb.2025.1628355)
Supplement: Supplementary file 1 [file Data_Sheet_1.PDF]

frontiers

Supplementary Material

Successional Patterns of Microbial Communities Across Various Stages of Leaf Litter Decomposition in Poplar Plantations

Contents

Tables: 8

Figures: 4

Figure S1 Environmental diagram of sampling site.

Figure S2 Leaf litter mass remaining at different stage.

Figure S3 Non-metric multidimensional scaling (NMDS) analysis of microbial communities at different stages of leaf litter degradation. (A) NMDS analysis of bacterial communities. (B) NMDS analysis of fungal communities.

Figure S4 Quantitative analysis of gene copy number in microbial communities during leaf litter degradation based on qPCR. The copy number of bacterial 16S rRNA gene (A) and fungal ITS gene (B). Different lowercase letters represent significant differences among different decomposition stages as determined by Tukey test at the 0.05 level of significance.

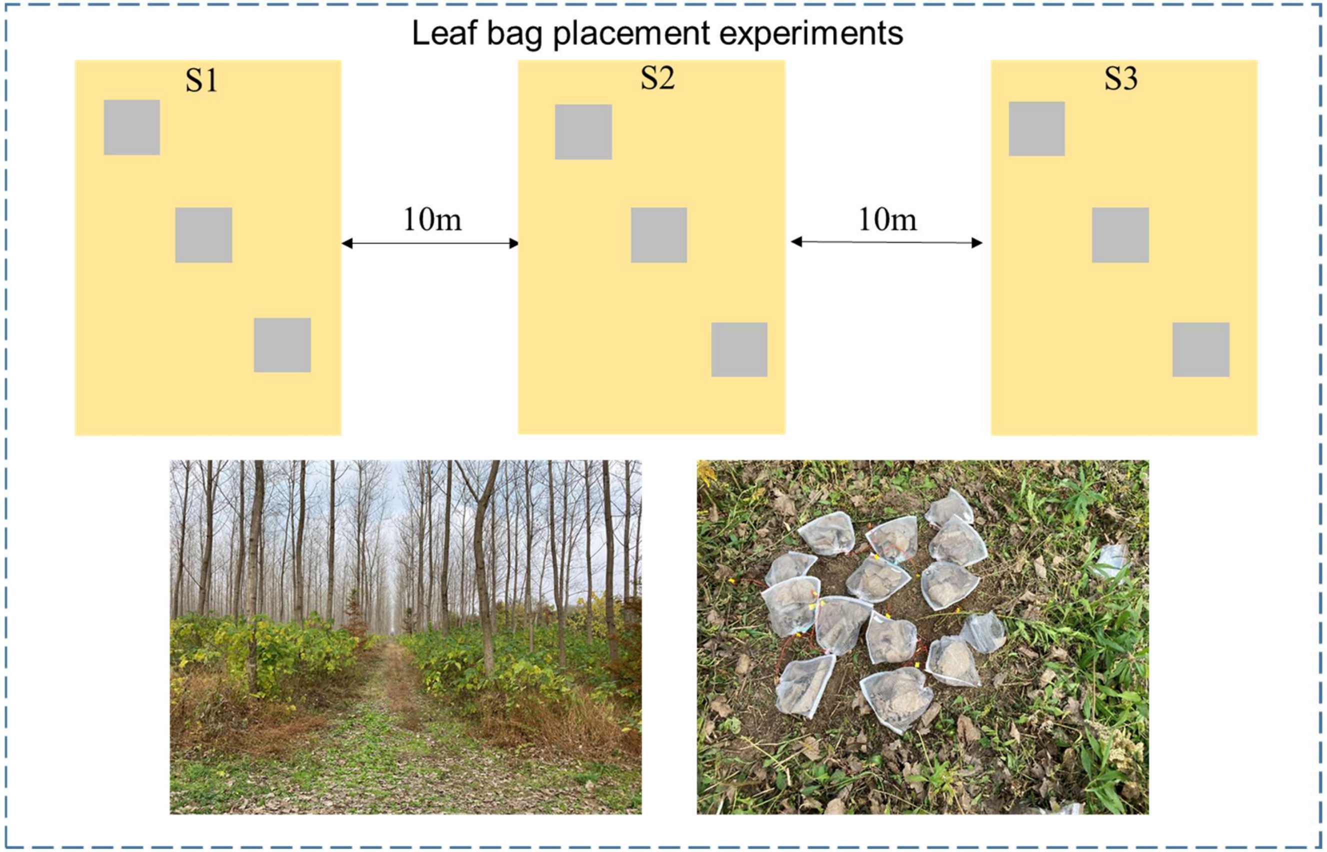

Fig. S1 Environmental diagram of sampling site.

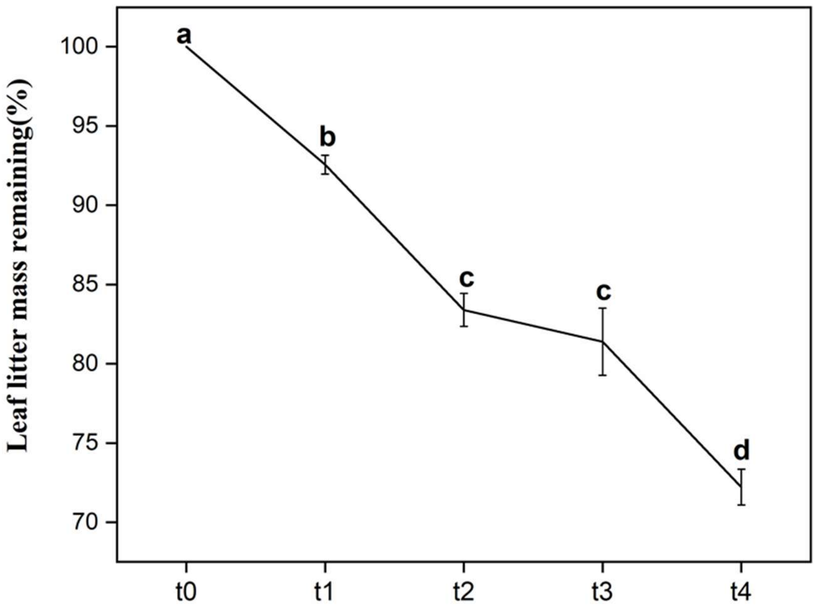

Fig. S2 Leaf litter mass remaining at different stage.

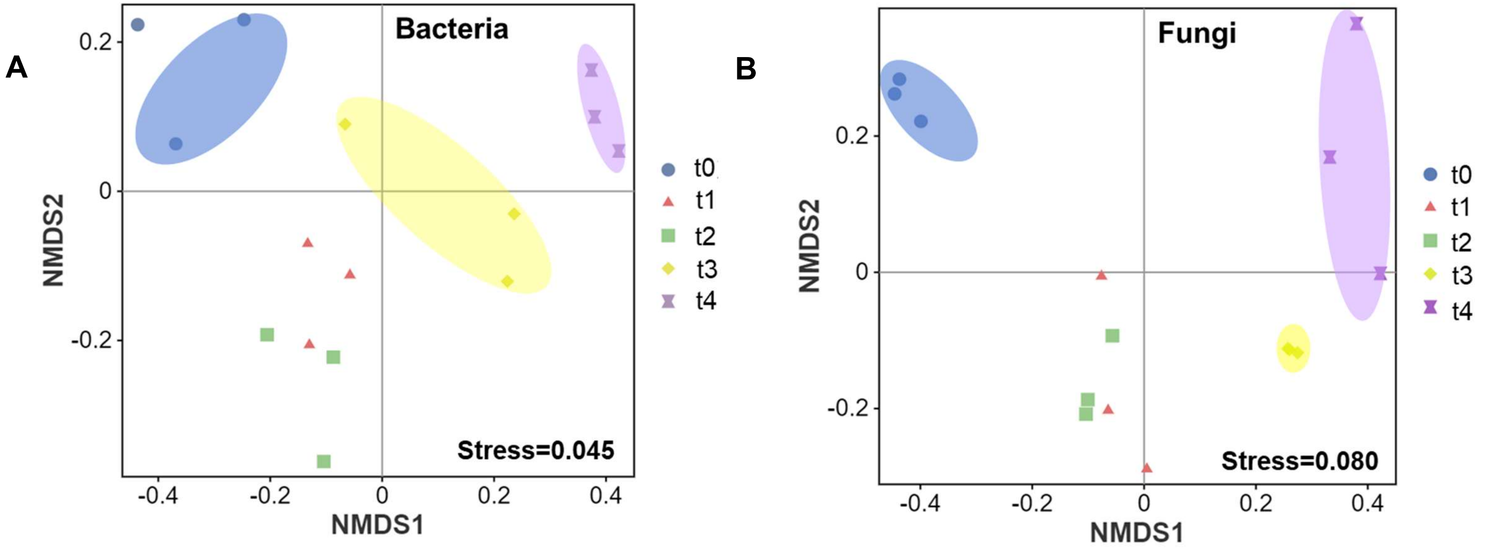

Fig. S3 Non-metric multidimensional scaling (NMDS) analysis of microbial communities at different stages of leaf litter degradation. (A) NMDS analysis of bacterial communities. (B) NMDS analysis of fungal communities.

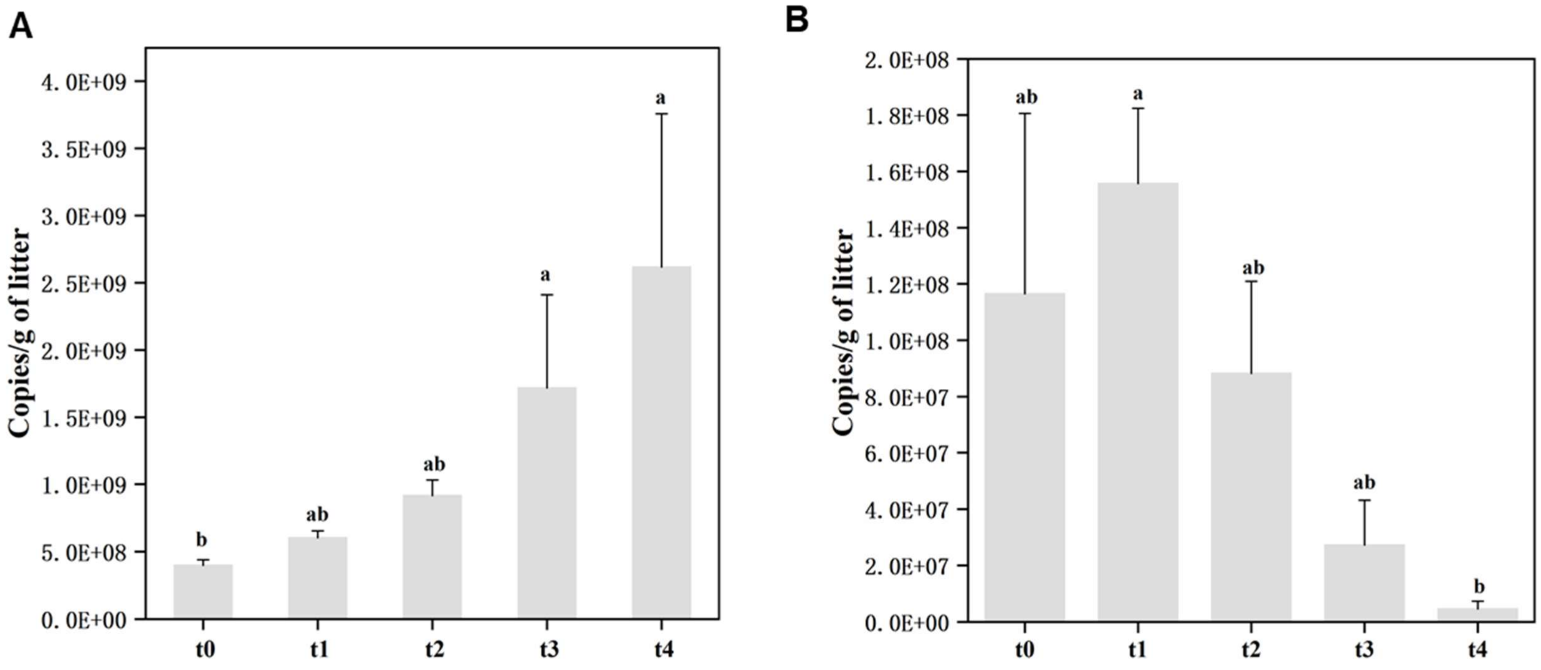

Fig. S4. Quantitative analysis of gene copy number in microbial communities during leaf litter degradation based on qPCR. The copy number of bacterial 16S rRNA gene (A) and fungal ITS gene (B). Different lowercase letters represent significant differences among different decomposition stages as determined by Tukey test at the 0.05 level of significance.

Table S1 The primer sequences of qPCR.

Target	Forward Primer 5'→3'	Reverse Primer 5'→3'
Bacteria 16S	EUB338: ACT CCT ACG GGA GGC AGC AG	EUB518R: ATT ACC GCG GCT GCT GG
Fungal ITS	ITS1f: TCC GTA GGT GAA CCT GCG G	5.8S: CGC TGC GTT CTT CAT CG
PUC-16S	CTCTAGAGGATCCCC ACT CCT ACG GGA GGC AGC AG	TCGAGCTCGGTACCC ATT ACC GCG GCT GCT GG
PUC-ITS	CTCTAGAGGATCCCC TCC GTA GGT GAA CCT GCG G	TCGAGCTCGGTACCC CGC TGC GTT CTT CAT CG

Table S2 Changes of litter chemical properties at different stages.

Variable		Stages				
		t0	t1	t2	t3	t4
Macronutrients	TC (g kg^{-1})	393.51±3.83a	378.62±2.78b	344.8±5.21c	336.27±14.76c	343.45±8.33c
	TN (g kg-1)	22.02±0.164c	20.94±0.148c	25.09±0.474b	25.78 ±0.566b	27.37±0.278a
	TP (g kg-1)	1.95±0.01a	1.72±0.032ab	1.53±0.171b	1.55±0.021b	1.59±0.028b
	TK (g kg-1)	6.08±0.14a	2.86±0.39b	2.66±0.10b	1.86±0.31b	2.37±0.32b
Compounds	Cellulose	100%±0.00%a	93%±0.15%b	79%±0.09%c	69%±0.95%d	58%±0.92%e
	Hemicellulose	100%±0.00%a	87%±0.15%b	86%±0.10%bc	84%±1.15%c	70±1.12%d
	Lignin (g kg-1)	238.62±11.94d	326.56±2.67a	294.27±6.20bc	313.90±3.61ab	290.9667±1.69c
Stoichiometric ratios	C/N	17.87±0.17a	18.08±0.13a	13.74±0.21b	13.04±0.57b	12.55±0.30b
	N/P	11.29±0.08d	12.15±0.09c	16.38±0.25b	16.66±0.30ab	17.21±0.14a
	Lignin/N	10.84±0.44c	15.59±0.10a	11.73±0.20b	12.18±0.11b	10.63±0.05c

Different letters indicate significant differences between stages through multiple comparisons using Tukey test at $p < 0.05$ level.

Table S3 The alpha diversity of microbial communities at different stages of litter degradation.

Community	Time	Chao1		Shannon	
		Value	Turkey	Value	Turkey
Fungi	t0	64.33±10.11	b	2.00±0.09	c
	t1	182.67±36.38	a	4.43±0.39	a
	t2	137.67±7.78	ab	4.16±0.09	a
	t3	214.33±13.48	a	3.39±0.54	ab
	t4	183.33±43.52	a	2.57±0.34	bc
Bacteria	t0	852±176.33	cd	6.28±0.21	cd
	t1	1071±72.74	bc	7.20±0.22	bc
	t2	499±88.25	d	5.62±0.38	d
	t3	1429.33±135.91	b	7.74±0.38	b
	t4	2295±76.99	a	8.94±0.05	a

Table S4 Relative abundance of phyllospheric bacterial community composition at the class level (relative abundance ⩾0.01%).

	t0	t1	t2	t3	t4	***F***	***P***
Actinobacteria	34.41±4.93	27.37±7.19	22.81±4.62	32.16±4.34	57.70±4.69	4.404	**0.026***
Alphaproteobacteria	39.47±10.26	23.31±2.16a	29.16±8.47a	47.48±7.13a	24.65±4.19a	1.431	0.293
Gammaproteobacteria	20.96±7.1	22.73±3.02ab	43.17±13.78a	9.72±1.03b	4.84±0.96b	2.919	0.077
Bacteroidia	1.34±0.41	24.83±4.96b	4.086±0.86b	4.76±1.50b	1.91±0.25b	11.625	**0.001****
Deltaproteobacteria	0.54±0.13	0.54±0.10b	0.09±0.02b	2.22±0.50a	1.99±0.23a	9.292	**0.002****
Thermoleophilia	0.22±0.09	0.12±0.007c	0.16±0.05c	1.20±0.21b	3.35±0.04a	116.856	**0.001****
Acidimicrobiia	0.07±0.02	0.064±0.02b	0.014±0.01b	0.43±0.17b	1.20±0.17a	13.99	**0.001****
Bacilli	0.46±0.26	0.093±0.06a	0.012±0.0052a	0.02±0.005a	0.08±0.04a	1.574	0.255
Chloroflexia	0.039±0.03	0.016±0.01b	0.00b	0.087±0.03b	0.46±0.17a	4.098	**0.032***
Longimicrobia	0.0034±0.003	0.00a	0.00a	0.25±0.16a	0.27±0.02a	2.702	0.092
Oxyphotobacteria	0.58±0.36	0.0043±0.0018a	0.0038a	0.001a	0.0036±0.002a	1.705	0.225

Different letters indicate significant differences between stages through multiple comparisons using Tukey test at $p < 0.05$ level.

Table S5 Analysis of variance of relative abundance of phyllospheric fungal community composition at the class level (relative abundance ⩾0.01%).

	t0	t1	t2	t3	t4	***F***	***P***
Dothideomycetes	92.60±1.28	64.08±3.16	57.75±3.33	26.27±3.07	77.96±8.34	2.61	0.10
Sordariomycetes	0.08±0.053	9.53±1.67	17.08±1.16	10.61±4.27	11.37±3.13	3.903	**0.037***
Leotiomycetes	0.0015±0.001	0.26±0.12	0.77±0.29	47.17±13.67	37.78±16.89	3.838	**0.038***
Tremellomycetes	0.093±0.062	16.97±6.36	20.76±2.99	0.11±0.018	0.010±0.008	7.267	**0.005****
Pucciniomycetes	6.94±1.02	0.63±0.46	3.08±1.88	0.011±0.0089	0.016±0.010	6.134	**0.009****
Agaricomycetes	0.002±0.0016	0.218±0.144	0.15±0.11	0.38±0.26	1.08±0.80	0.708	0.553
Cystobasidiomycetes	0.0061±0.002	0.30±0.11	0.18±0.02	0.022±0.057	0.049±0.04	3.628	**0.045***
Eurotiomycetes	0.00	0.028±0.02	0.0037±0.003	0.027±0.01	0.19±0.07	3.706	**0.042***
Ustilaginomycetes	0.080±0.017	0.016±0.011	0.006±0.0026	0.005±0.003	0.00b	1.139	0.393
Exobasidiomycetes	0.051±0.019	0.0021±0.0017	0.00	0.00	0.00b	1.461	0.285
Agaricostilbomycetes	0.00	0.0174±0.007	0.00	0.049±0.018	0.024±0.005	1.772	0.211
Orbiliomycetes	0.00	0.00	0.00	0.00	0.11±0.017	27.706	**0.001****

Different letters indicate significant differences between stages through multiple comparisons using Tukey test at $p < 0.05$ level. * indicates $P<0.05$, and ** indicates $P < 0.01$.

Table S6 Analysis of variance of relative abundance (⩾0.01%) of phyllospheric bacteria community composition at the genus level (Top 30).

	t0	t1	t2	t3	t4	***F***	***P***
Allorhizobium-Neorhizobium-Pararhizobium-Rhizobium	10.10±4.27	12.95±2.97	19.15±7.37	15.62±8.14	2.56±0.78	0.887	0.506
Massilia	0.53±0.23	9.91±3.14	36.45±13.83	3.56±1.61	0.96±0.30	3.726	**0.042***
Actinoplanes	0.32±0.18	5.05±3.41	0.113±0.08	10.23±3.36	13.13±2.47	3.907	**0.037***
Sphingomonas	10.93±4.73	2.87±0.47	5.76±1.35	7.01±1.43	2.71±0.43	1.435	0.292
Flavobacterium	0.36±0.20	17.73±4.50	1.91±0.35	3.61±1.30	1.11±0.23	7.897	**0.004****
Pseudomonas	12.0±4.05	4.52±1.20	1.50±0.16	0.70±0.35	0.27±0.078	4.404	**0.026***
Nocardioides	3.66±1.90	1.23±0.36	0.92±0.32	5.061±0.55	7.66±0.88	5.303	**0.015***
Kineosporia	0.35±0.10	4.29±2.65	0.34±0.24	1.68±0.23	2.78±0.54	1.277	0.342
Aureimonas	6.69±4.30	0.38±0.04	0.89±0.16	2.59±0.70	0.38±0.078	1.267	0.345
Kineococcus	6.15±1.28	0.89±0.51	0.83±0.02	0.33±0.25	0.0074±0.0031	11.027	**0.001****
Devosia	0.21±0.08	0.86±0.17	0.16±0.072	3.66±0.59	2.57±0.53	12.069	**0.001****
Curtobacterium	0.57±0.19	2.30±1.00	5.09±1.91	0.26±0.062	0.29±0.21	3.011	0.072
Pseudokineococcus	6.04±2.27	0.43±0.23	0.21±0.66	0.041±0.03	0.00	4.426	**0.026***
Quadrisphaera	5.96±0.40	0.18±0.11	0.15±0.026	0.018±0.003	0.024±0.0067	134.514	0.001
Methylobacterium	3.72±2.20	0.61±0.17	1.07±0.33	0.80±0.20	0.43±0.17	1.223	0.361
Chryseobacterium	0.55±0.33	5.02±0.49	1.56±0.29	0.37±0.20	0.083±0.033	29.271	**0.001****
Mycobacterium	0.075±0.03	0.190±0.06	0.0202±0.009	0.77±0.13	2.02±0.09	78.226	**0.001****
Novosphingobium	0.30±0.01	1.01±0.12	0.69±0.21	2.73±0.24	0.47±0.083	26.299	**0.001****
Streptomyces	0.0059±0.002	0.130±0.10	0.00	0.092±0.05	2.08±0.75	4.682	**0.022***
Aeromicrobium	0.095±0.067	0.28±0.11	0.84±0.24	2.68±0.59	1.22±0.50	5.220	**0.016***
Microbacterium	1.52±0.076	0.67±0.10	0.34±0.048	0.70±0.11	0.70±0.17	1.024	0.441
Geodermatophilus	4.21±0.91	0.20±0.12	0.24±0.026	0.088±0.022	0.0162±0.007	3.792	**0.040***
Variovorax	0.21±0.10	0.40±0.06	0.49±0.098	1.22±0.35	0.47±0.133	3.076	0.068
Roseomonas	2.81±1.93	0.29±0.033	0.379±0.10	0.44±0.13	0.23±0.068	1.090	0.412
Rhodopseudomonas	0.033±0.01	0.34±0.19	0.11±0.079	2.31±0.48	1.29±0.094	3.395	0.053
Delftia	3.98±1.28	0.014±0.007	0.022±0.0036	0.0075±0.0037	0.021±0.005	3.328	0.056
Stenotrophomonas	0.096±0.047	1.05±0.23	0.18±0.041	0.85±0.215	0.11±0.025	0.492	0.742
Actinophytocola	0.031±0.01	0.00	0.00	0.00	3.41±1.04	7.097	**0.006****
Tardiphaga	0.00	0.0072±0.004	0.030±0.020	2.41±1.02	0.68±0.074	1.691	0.228
Caulobacter	0.015±0.005	0.14±0.02	0.022±0.016	1.56±0.06	0.35±0.064	4.574	**0.023***

Different letters indicate significant differences between stages through multiple comparisons using Tukey test at $p < 0.05$ level. * indicates $P<0.05$, and ** indicates $P < 0.01$.

Table S7 Analysis of variance of relative abundance (⩾0.01%) of phyllospheric fungal community composition at the genus level (Top 30).

	t0	t1	t2	t3	t4	***F***	***P***
Ramularia	81.42±3.22	9.36±3.32	4.74±0.74	0.035±0.002	0.0023±0.002	186.601	**0.001****
Papiliotrema	0.049±0.03	16.20±5.92	20.04±3.06	0.063±0.02	0.0032±0.002	7.494	**0.005****
Alternaria	0.378±0.16	10.43±2.85	11.14±1.63	3.41±0.51	0.36±0.18	8.433	**0.003****
Acremonium	0.0086±0.004	1.28±0.183	13.00±2.60	1.46±0.70	0.76±0.57	13.037	**0.001****
Cladosporium	0.13±0.024	3.66±1.86	3.80±0.81	2.85±0.20	0.59±0.23	2.376	0.122
Melampsora	6.94±1.02	0.63±0.46	3.08±1.88	0.011±0.009	0.016±0.01	6.134	0.009**
Pseudocercospora	7.81±4.14	0.22±0.08	0.23±0.15	0.023±0.007	0.021±0.008	2.296	0.131
Allophoma	0.014±0.01	0.171±0.08	0.44±0.09	2.21±1.44	1.62±1.19	0.700	0.610
Leptospora	0.002±0.0016	1.40±0.63	0.70±0.23	0.84±0.22	0.0947±0.05	2.180	0.145
Sphaerellopsis	1.62±1.29	0.078±0.03	1.17±0.51	0.022±0.018	0.00	1.014	0.445
Sarocladium	0.003±0.002	0.43±0.33	0.22±0.07	1.20±0.67	0.40±0.13	1.16	0.384
Plectosphaerella	0.021±0.017	0.083±0.04	0.0067±0.003	1.02±0.10	0.72±0.55	2.365	0.123
Pyrenochaeta	0.00	0.005±0.004	0.0026±0.002	1.48±1.17	0.068±0.03	1.03	0.438
Didymella	0.00	0.027±0.015	0.81±0.065	0.43±0.15	0.14±0.10	9.937	0.002
Podospora	0.00	0.0016±0.0013	0.00	0.00b	1.39±0.72	2.477	0.112
Acrocalymma	0.001±0.0008	0.90±0.48	0.071±0.06	0.05±0.006	0.026±0.01	2.133	0.151
Bulleromyces	0.013±0.008	0.56±0.33	0.46±0.12	0.0145±0.004	0.00	2.018	0.168
Stagonosporopsis	0.00	0.019±0.007	0.015±0.012	0.8348±0.378	0.10±0.06	2.985	0.073
Roussoella	0.00	0.025±0.02	0.0047±0.0038	0.053±0.01	0.85±0.66	1.047	0.431
Septoria	0.62±032a	0.097±0.02	0.104±0.016	0.010±0.005	0.003±0.002	2.077	0.159
Farlowiella	0.00a	0.00	0.00	0.73±0.59	0.009±0.007	1.018	0.444
Pyrenochaetopsis	0.00a	0.017±0.009	0.026±0.011	0.18±0.06	0.31±0.23	1.009	0.447
Lophiostoma	0.00a	0.057±0.047	0.072±0.056	0.135±0.06	0.19±0.14	0.637	0.648
Neodevriesia	0.052±0.0032a	0.039±0.01	0.015±0.012	0.106±0.044	0.14±0.06	1.436	0.292
Diaporthe	0.00a	0.22±0.18	0.018±0.01	0.054±0.03	0.0032±0.003	0.855	0.522
Paraphoma	0.00a	0.14±0.11	0.0047±0.0034	0.15±0.045	0.009±0.003	1.342	0.321
Stagonospora	0.00b	0.18±0.088	0.077±0.012	0.012±0.003	0.00	2.985	0.073
Fusarium	0.00a	0.00	0.00	0.00	0.29±0.23	1.00	0.452
Clonostachys	0.00a	0.15±0.11	0.00	0.052±0.02	0.078±0.016	1.013	0.446
Periconia	0.0015±0.0012b	0.0624±0.02	0.064±0.02	0.141±0.03	0.0021±0.0017	6.623	**0.007****

Different letters indicate significant differences between stages through multiple comparisons using Tukey test at $p < 0.05$ level. * indicates $P<0.05$, and ** indicates $P < 0.01$.

Table S8 Properties of co-correlation networks of microbial community during litter decomposition.

**Community**	**Total nodes**	**Total links**	**Positive correlation**	**Average Degree**	**Average Path Length**	**Average Clustering Coefficient**	**Modularity (no. of modules)**
Bacteria	372	1363	99.49%	7.328	3.496	0.607	0.667 (66)
Fungi	56	58	100%	2.071	3.698	0.388	0.788 (14)
